# Transcatheter Tricuspid Valve Replacement: Principles and Design

**DOI:** 10.3389/fcvm.2018.00129

**Published:** 2018-09-19

**Authors:** Ozan M. Demir, Damiano Regazzoli, Antonio Mangieri, Marco B. Ancona, Satoru Mitomo, Giora Weisz, Antonio Colombo, Azeem Latib

**Affiliations:** ^1^Interventional Cardiology Unit, Cardio-Thoracic-Vascular Department, San Raffaele Scientific Institute, Milan, Italy; ^2^Department of Cardiology, Hammersmith Hospital, Imperial College Healthcare NHS Trust, London, United Kingdom; ^3^Department of Cardiology, Montefiore Medical Center, New York, NY, United States; ^4^Division of Cardiology, Department of Medicine, University of Cape Town, Cape Town, South Africa

**Keywords:** tricuspid regurgitation, valve replacement, cardiac imaging, tricuspid valve, structural heart disease

## Abstract

Tricuspid regurgitation (TR) may affect as much as 65–85% of the population with the prevalence of moderate-to-severe TR in the United States reported at greater than 1.6 million. However, only 8,000 tricuspid valve operations are performed annually in the United States. As severe TR is associated with poor outcomes, there is an unmet clinical need for surgical or percutaneous transcatheter based treatment of TR. Over the last two decades there have been significant developments in percutaneous transcatheter based therapies for valvular disease. However, this progress has not been mirrored for the tricuspid valve until recently; we are now at a cross-roads of new transcatheter devices becoming available for treatment of TR. In this review, we discuss the principles of performing transcatheter tricuspid valve replacement, analyze the devices that can be utilized and outline the challenges related to this procedure.

## Introduction

Tricuspid regurgitation (TR) is a commonly encountered manifestation of valvular heart disease, it may affect as much as 65–85% of the population (1, 2). The majority of these are no more than mild TR which is deemed non-pathological and a normal variant, however, moderate-to-severe TR is usually pathological and associated with poor prognosis (3). The etiology of TR can be divided into primary (organic) and secondary (functional), in relation to the presence of structural abnormalities of the tricuspid valve (TV) apparatus. Approximately 80% of significant TR is functional (FTR), occurring due to annular dilation and subsequent leaflet tethering causing malcoaptation (4, 5). Organic TR can be either congenital or acquired. Congenital primary TR may arise due to Ebstein's anomaly, atrioventricular defects and myxomatous prolapse. Acquired primary TR can occur due to endocarditis, rheumatic disease, carcinoid, flail leaflets caused by trauma, or from pacemaker lead implantation (6). Patients with TR often experience clinical symptoms of right-sided heart failure, including dyspnea, restriction of functional capacity, frequent hospitalization, liver, and kidney failure.

The prevalence of moderate-to-severe TR in the United States has been reported at greater than 1. 6 million. Despite this only 8,000 TV operations are performed annually in the United States (7). Furthermore, with increasing severity of TR, 1-year mortality increases, reaching greater than 36% in those with severe TR (3). Hence, there is an unmet clinical need for surgical or percutaneous treatment of TR. Over the last two decades, there has been significant developments in transcatheter based therapies for valvular disease. However, this progress has not been mirrored for the TV until recently; we are now at a cross-roads of new transcatheter devices becoming available for treatment of TR. In this review, we discuss the principles of performing transcatheter tricuspid valve replacement (TTVR), analyze the devices that can be utilized and outline the challenges related to this procedure.

## Principles of transcatheter tricuspid valve replacement

The development of devices specifically designed for percutaneous TV repair or replacement are currently at an early stage. In this section, we will analyze the main challenges of TTVR procedures, in order to become a safe and effective alternative to medical therapy or high-risk surgical interventions.

### Tricuspid valve anatomy and technical challenges

The TV apparatus is a complex structure consisting of 3 leaflets (anterior, posterior and septal) that are inserted to the tricuspid annulus and attached through the chordae tendinae to the papillary muscles of the right ventricle (RV). The tricuspid annulus is relatively less fibrous when compared with the mitral valve and the right coronary artery surrounds the parietal attachment of the valve. The normal physiological valve is a dynamic, nonplanar structure that varies in size and shape throughout the cardiac cycle (8).

**Figure 1:**
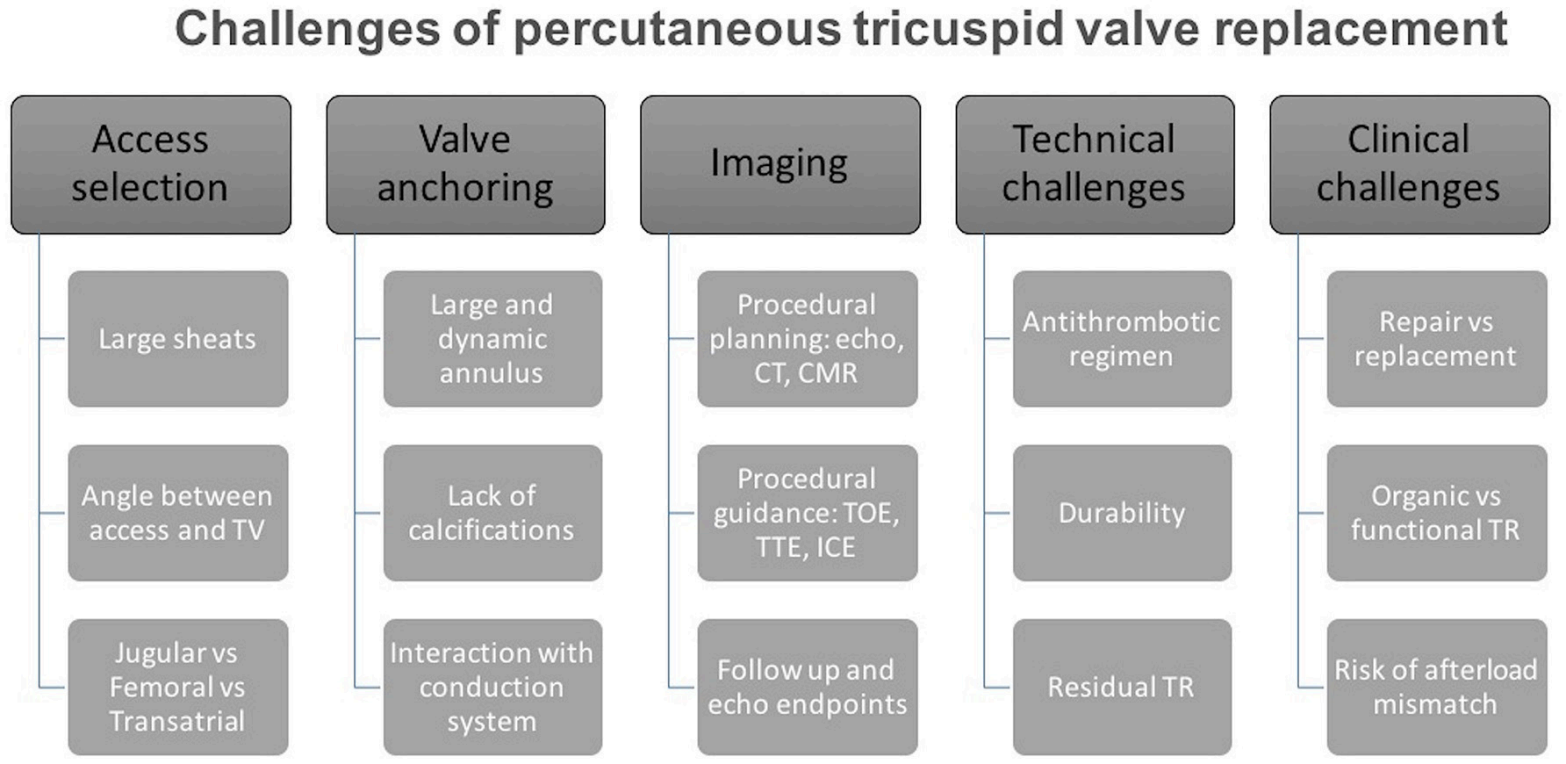
Challenges of percutaneous tricuspid valve replacement. Systematic diagram outlining main challenges that will be encountered during percutaneous tricuspid valve replacement.

This anatomical and functional complexity reflects on different procedural issues that must be taken in account during the development of tricuspid prosthesis and relative delivery systems (Figure 1):

- *Access selection:* One of the main anatomical challenges for current generation TTVR devices is the dimensions of the TV annulus; the TV annulus is large (larger than mitral) and it gets even larger when longstanding FTR and RV dilation are present. Hence, prosthetic TV valves have a large profile requiring large caliber sheaths. Large bore venous access (up to 45 Fr, with current devices) is of paramount importance when planning a replacement procedure and led to the preferential selection of three potential sites, that have potential advantages and disadvantages (9). Trans-jugular access, obtained either percutaneously or surgically, offers a good angle to approach the TV, with a delivery system that requires less steering, but requires a vein large enough to accommodate the sheath without being damaged. The femoral vein is the safest access route due to its size, but the angle between the inferior vena cava and TV is steep and may hamper the procedure. Trans-atrial approach requires a surgical approach (anterior right thoracotomy) but allows direct management of the access site. There is no clear answer on which of these is the better access route, although a percutaneous route will be essential for success.
- *Valve anchoring:* The tricuspid annulus is not calcific, is a three dimensional and dynamic structure; therefore, the possibility to achieve good prosthesis fixation and stability is a major issue. While no clear data is currently available, it can be hypothesized that a large prosthesis in tricuspid position may need a great capability of adaptation to the aforementioned anatomical characteristics: self-expanding devices may be more effective and with lower risk of annular stretching and damage. On the other hand, the TV prosthesis is associated with a lower risk of outflow tract obstruction as compared with mitral valve and active grasping of the native tricuspid leaflets may be not needed (10). However, at present valve anchoring is still a major unanswered issue.
- *Interaction with conduction system and with pacing devices:* The atrioventricular (AV) node lies in the muscular portion of the atrio-ventricular septum, near the ostium of the coronary sinus (at the apex of the triangle of Koch). The bundle of His is a direct continuation of the AV node and it passes through the right trigone of the central fibrous body to reach the ventricular septum. This area is near to the commissure between septal and anterior tricuspid leaflets (11). This close relationship between the tricuspid structure and the conduction system may be an issue when planning TTVR. In fact, surgical annuloplasty with dedicated tricuspid rings is often incomplete in order to avoid placing stitches in the septal area to reduce the incidence of complete AV block and subsequent pacemaker implantation. Percutaneously implanted bioprosthesis will likely not be able to avoid stretching that area. The incidence of rhythm disturbances is therefore expected to be higher than with repair, eventually leading to a second major issue: how to manage pacemaker devices during valve implantation. Indeed, prosthesis deployment may lead to a dislodgment of a pre-existing ventricular lead and, on the other hand, the prosthesis itself may hamper PM implantation.
- *Antithrombotic regimen:* No evidence is available on the selection of antithrombotic regimen specifically following TTVR (12). However, considering the low flow on the right-side of the heart and the size of the TTVR prosthesis, we would recommend life-long anticoagulant therapy in all patients with many patients already having an indication for anticoagulation, e.g., atrial fibrillation.
- *Durability:* Concerns regarding structural valve degeneration remains an important drawback of surgical and transcatheter bioprostheses (13). There is scarcity of evidence regarding the durability of bioprostheses in the tricuspid position however data from early experience are reassuring whilst we await long-term outcomes. In comparison, currently there is no data on TTVR durability. Hence, this will be a major issue when percutaneous treatment of the TV expands from compassionate cases to younger and lower risk patients with organic or functional tricuspid regurgitation.
- *Residual regurgitation:* The management of residual regurgitation after TTVR will be a major issue. The quantification of tricuspid valve regurgitation is still debatable and there is no clear threshold that is considered prognostic (14). The identification and quantification of residual insufficiency after TTVR is expected to be even more complex, and will require a comprehensive multi-parameter approach. Furthermore, since the TV annulus is saddle-shaped and has considerable systo-diastolic modification of its diameter, we can expect some type of incomplete apposition of the prosthetic valve, especially in the septal portion of the native annulus (11).
- *Acute increase in RV afterload:* Whilst tricuspid-valve replacement may eliminate the problem of residual regurgitation it can result in acute increase the right ventriclar afterload. In addition, RV dysfunction is common in patients with TR, either subtle or overt (15, 16) and is associated with adverse outcomes (17, 18). In the presence of RV dysfunction, it is assumed that abrupt discontinuation of the tricuspid regurgitation is associated with significant increase of the RV afterload. The RV is very sensitive to afterload changes (19). Thus, successful TV replacement may result in acute RV decompensation and adverse outcomes.

## Patient selection

A fundamental principle of all invasive procedures is patient selection. Surgical TV repair is the most common form of TV surgery and is predominantly performed concomitantly with another major surgical procedure (e.g., mitral valve surgery). Surgical TV repair is seldom performed for isolated TV disease (13% of surgical cases) (20). Furthermore, surgery for TR can be associated with a high risk of morbidity and mortality, with perioperative mortality reaching up to 10% in selected cases (1, 21). For these patients, new percutaneous transcatheter approaches may address this unmet clinical need. At present, percutaneous TV procedures may be considered in patients with severe TR who remain symptomatic despite optimal medical therapy. While various devices that mimic surgical repair have been evaluated in the last few years in clinical studies or for compassionate use, percutaneous valve replacement is still in an early stage (22). Similar to what is occurring with transcatheter mitral valve procedures, the parameters that may lead operators to prefer a valve replacement over a safe (although maybe less effective) repair procedure are still debatable. While there is still no clear evidence about this issue, we believe that reparative procedures may be less effective in the following subset of patients:

- TTVR may be preferred over repair techniques in those patients in where mechanism of TR is not functional. Fibrotic leaflets (as a result of carcinoid syndrome or of rheumatic disease) or large leaflet prolapse may be suitable of TTVR over repair.
- On the other hand, patients with extremely dilated annuli and/or with extreme leaflet tethering have low probability to be successfully treated with currently available repair devices unless annular and leaflet devices are combined. In these patients, tricuspid valve replacement may be the first option in order to offer the best result.
- Lastly, a fundamental aspect is pre-procedural evaluation of pulmonary hemodynamics and RV function: despite the lack of evidence, we can hypothesize that an acute reduction in TR in patients with severe RV dysfunction and severe pulmonary hypertension may result in acute afterload mismatch despite technical and procedural success (23). Ventricular afterload mismatch, defined as acute impairment of systolic function after mitral valve repair or replacement (both surgical or percutaneous), is well known in mitral valve surgery and is a major issue in patients with functional mitral regurgitation and reduced ejection fraction. While percutaneous approaches seem to reduce the risk and the severity of this phenomenon by avoiding factors like the effects of open-heart surgery, cardiopulmonary bypass, and cardioplegic arrest, its incidence is estimated to be about 25% in patients treated with MitraClip (24). The RV is less able to tolerate acute changes in pressure, therefore the incidence and severity of afterload mismatch may be even higher, and this may hamper the acute efficacy of TTVR. Moreover, our ability to identify predictors of acute RV dysfunction remains poor (24). Indeed, the RV has a unique crescent shape, which adds complexity to the quantification of its size and function, thus making the effect of acute afterload increase less predictable. Furthermore, the treatment of left ventricular afterload mismatch is based on inotropic agents and arterial vasodilators; this class of drugs is known to be much less effective on the pulmonary circulation. All these aspects must be taken in account when planning TV procedures and may lead to patients with advanced RV dysfunction being excluded (or to prefer repair over replacement). In addition, these patients may require post-procedural support with inotropic agents or mechanical support in order to avoid organ failure and, eventually, death.

## Imaging for transcatheter tricuspid valve replacement

**Figure 2:**
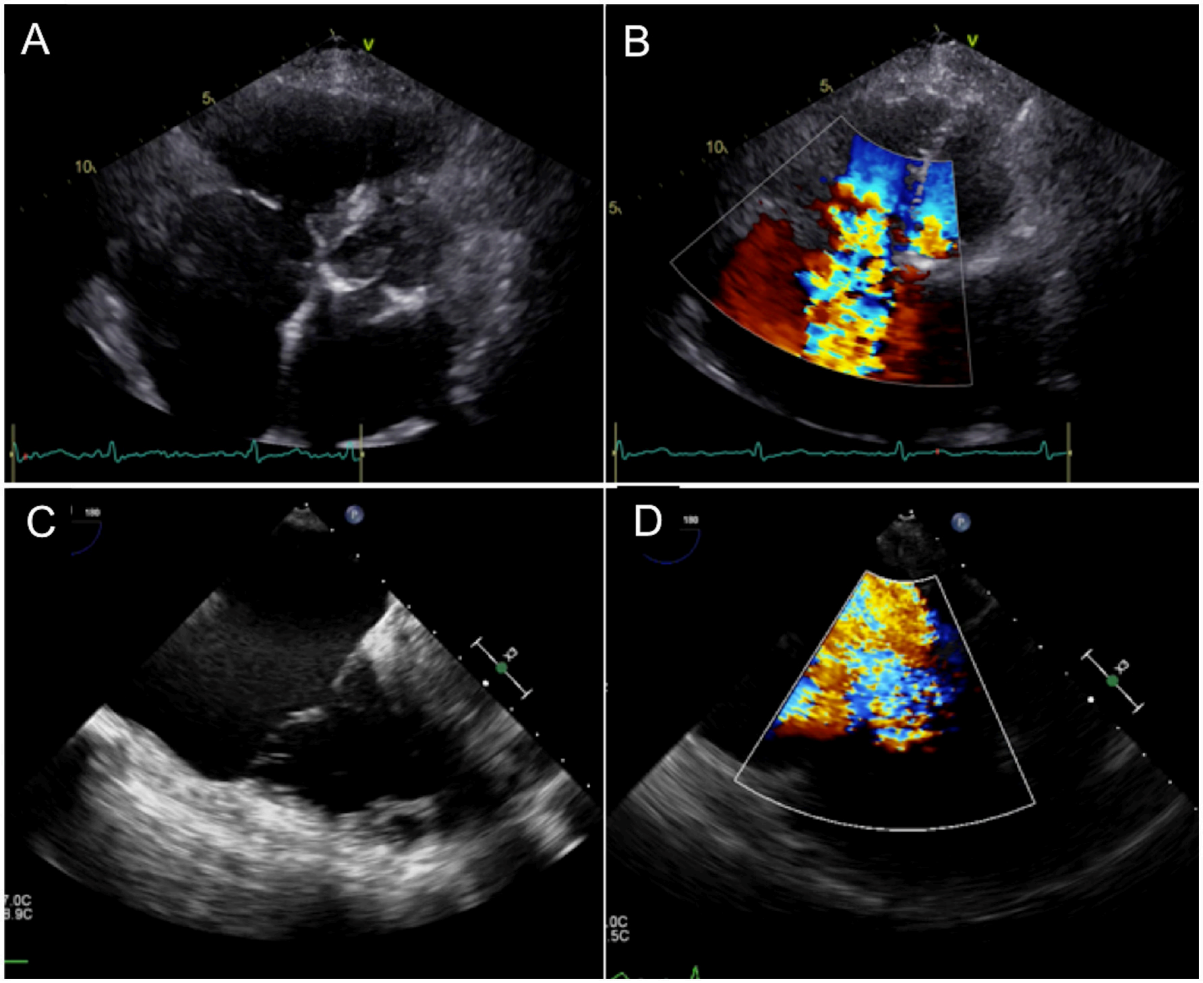
Echocardiographic Evaluation of Severe Tricuspid Regurgitation and its etiology. **(A,B)** Extreme tethering and annular dilatation with loss of coaptation, resulting in severe valvular regurgitation. **(C,D)** Large posterior leaflet prolapse with severe eccentric regurgitant jet.

**Figure 3:**
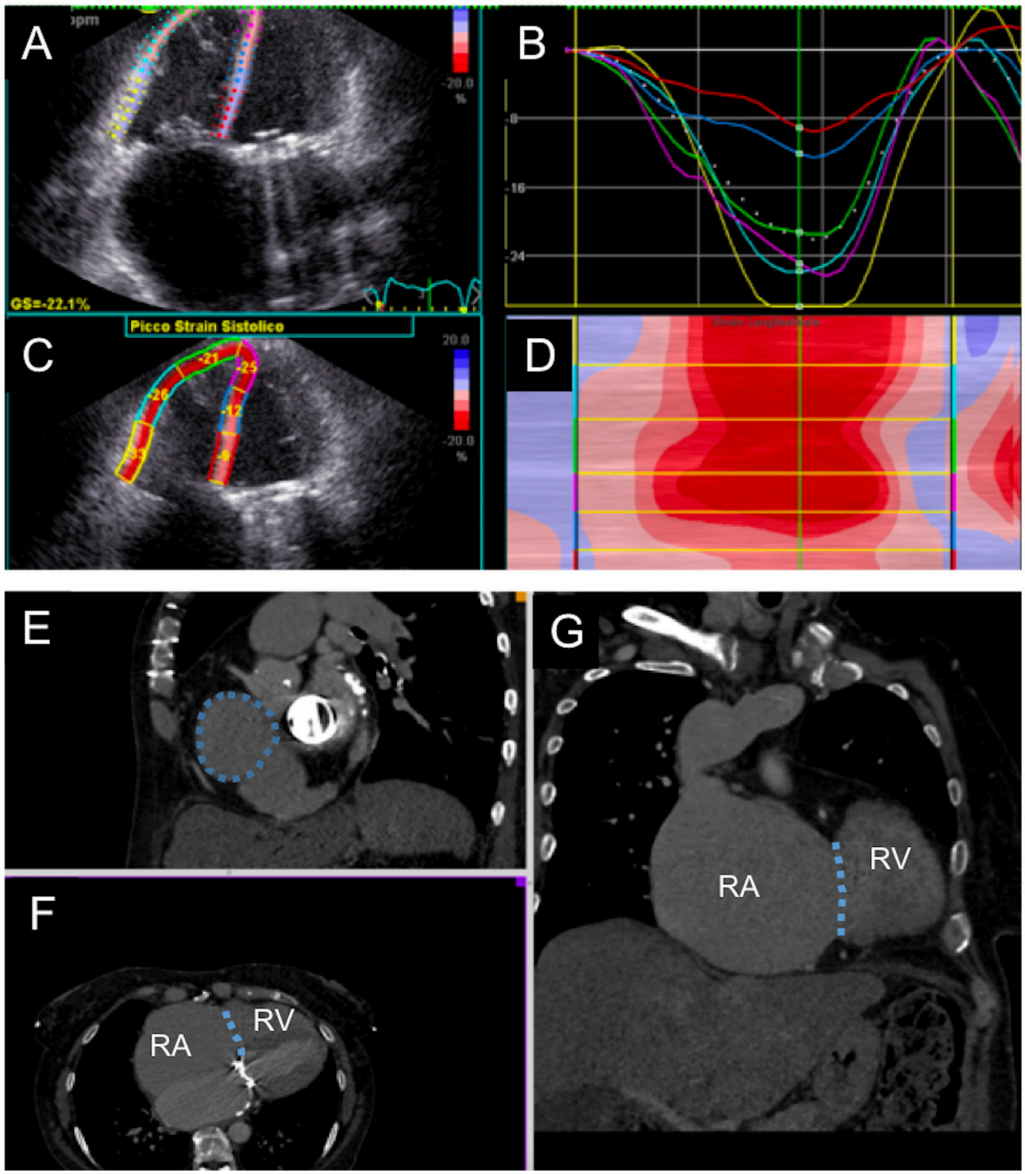
Multimodality evaluation for percutaneous tricuspid valve replacement. **(A–D)** Echocardiographic evaluation of right ventricular function with semi-automated strain measurement. **(E–G)** Computed tomography cardiac chamber and tricuspid valve evaluation; the patient presented with severe right ventricular (RV) and right atrial (RA) dilatation; tricuspid annulus is highlighted with dotted line.

Similar to mitral valve procedures, integration of interventional and imaging techniques is essential for TTVR. Cardiac imaging is essential at three crucial points: Pre-procedural planning (TR diagnosis, grading and etiology), interventional guidance, and follow-up after the procedure. The main imaging modalities are echocardiography (transthoracic, transesophageal, 3D echocardiography and intracardiac echo; Figure 2) (8), computed tomography (CT) imaging, and cardiac magnetic resonance (CMR). In this section the role of different types of imaging during the phases of tricuspid valve replacement will be discussed (Figure 3).

### Tricuspid regurgitation evaluation and pre-procedural planning

TTVR requires accurate evaluation of the TV apparatus (with a particular focus on the TV annulus) and of the RV.

- Echocardiography plays a major role, allowing complete assessment of the TV as well as RV pathophysiology. Since the TV is the most anterior structure of the heart, transthoracic 2D and 3D are fundamental and usually offer good imaging due to low thoracic impedance. It allows good evaluation of TR severity and etiology, as well as RV function and pulmonary pressure. Transesophageal echo (TEE), especially with full 3D sets, may help defining the mechanism of TR and morphologically characterize the TV: detailed assessment of tricuspid leaflet morphology and function can be obtained, as well as TV annulus dimensions and function.
- Computed tomography imaging, with multiplanar 3-dimensional reconstruction, is essential for preprocedural TV structural evaluation. It allows precise measurement of the RV dimensions and TV annulus size, relation with right coronary artery and the distance between the TV annulus and RV apex. In addition, CT enables preprocedural fluoroscopic angulation calculation and assessment of the access site dimensions and course (e.g. subclavian and axillary veins) for TTVR (25). However, CT exposes patients to iodinated contrast media, that need to be considered during procedural planning in those patients with impaired renal function.
- Cardiac magnetic resonance imaging can be utilized as adjunctive imaging modality for TV assessment prior to TTVR. It can be used for anatomical and functional assessment due to its excellent spatial resolution. CMR imaging with dedicated RV planes provides detailed RV chamber evaluation that is comparable to 3-dimensional echocardiography; however unlike echocardiography it is not hampered by patient's body habitus or lung fields. In addition, for patients with atrial tachycardias or fibrillation, free-breathing CMR sequencing is effective in providing quantitative evaluation. CMR evaluation of TV leaflet morphology can be challenging because of the thin nature of the leaflets. Evaluation of the TV annulus can be performed using breath-held cine using multiple long-axis views. Moreover, offline multiplane reconstruction can be performed to get detailed anatomical evaluation of the TV annulus. Lastly, severity of TR can be calculated using indirect quantification (difference between the planimetered RV stroke volume and forward pulmonic flow volume) (25).

### Procedural guidance

Multimodality imaging is essential for percutaneous TV interventions. Identification of TV annulus, for instance, can be performed with echocardiography but a guidewire placed into right coronary artery may be a useful angiographic marker. Due to its anterior location, the TV is not always well visualized with TEE; however, this remains the first-choice procedural imaging modality. When in doubt, transthoracic echo may be useful. The role of intracardiac echocardiography is not clear yet, but it may overcome the limitations of TEE and may be useful in ensuring coaxial alignment of the valved-stent to the TV annulus before deployment (26).

### Post-procedural follow-up

Once again, echocardiography has a key role in evaluation of procedural outcome. Echocardiography is able to evaluate not only the entity of regurgitation after TTVR that may be related to paravalvular or intravalvular jets, but also (and more importantly) the consequences of the insufficiency: RV reverse remodeling, regression of right atrial volume and inferior vena cava size may be used as surrogate endpoints of the success of the procedure (along with decrease in cardiac biomarkers like proBNP, creatinine, BUN, bilirubin and other liver enzymes). Postprocedural CT evaluation may be useful if there is uncertainty at follow-up echocardiography with regards to complications (e.g., device failure, detachment, leak, thrombosis) (26). The role of cardiac magnetic resonance is not clear yet, but we may hypothesize that it may be useful in those situations in which residual regurgitation grading is not clear.

### Recommended standard imaging

Pre-procedural echocardiographic assessment including TEE and CT imaging evaluation are fundamental. Echocardiography enables hemodynamic and anatomical TV evaluation, and CT imaging permits precise device sizing and estimation of fluoroscopic angulations for the procedure. Intra-procedurally, fusion imaging (echocardiography and fluoroscopy) facilitates improved understanding of anatomical structures while showing enhanced visualization of catheter and device movements. In particular, it has a role in patients where technical difficulty is encountered and during initial learning curve implanting TTVR. Furthermore, in cases with poor transthoracic and transesophageal window we have found intra-cardiac echocardiography particularly helpful. It can be helpful for navigation inside right heart chambers, to visualize the device, to guide their fine positioning and orientation (coaxialization), and to discern the annulus from the leaflets.

## Transcatheter tricuspid valve replacement devices

While many companies are working on percutaneous tricuspid valve replacement devices, currently only a few have been successfully implanted in humans and there are a number of devices in development. The following section provides a brief overview of these devices.

### NaviGate prosthesis

**Figure 4:**
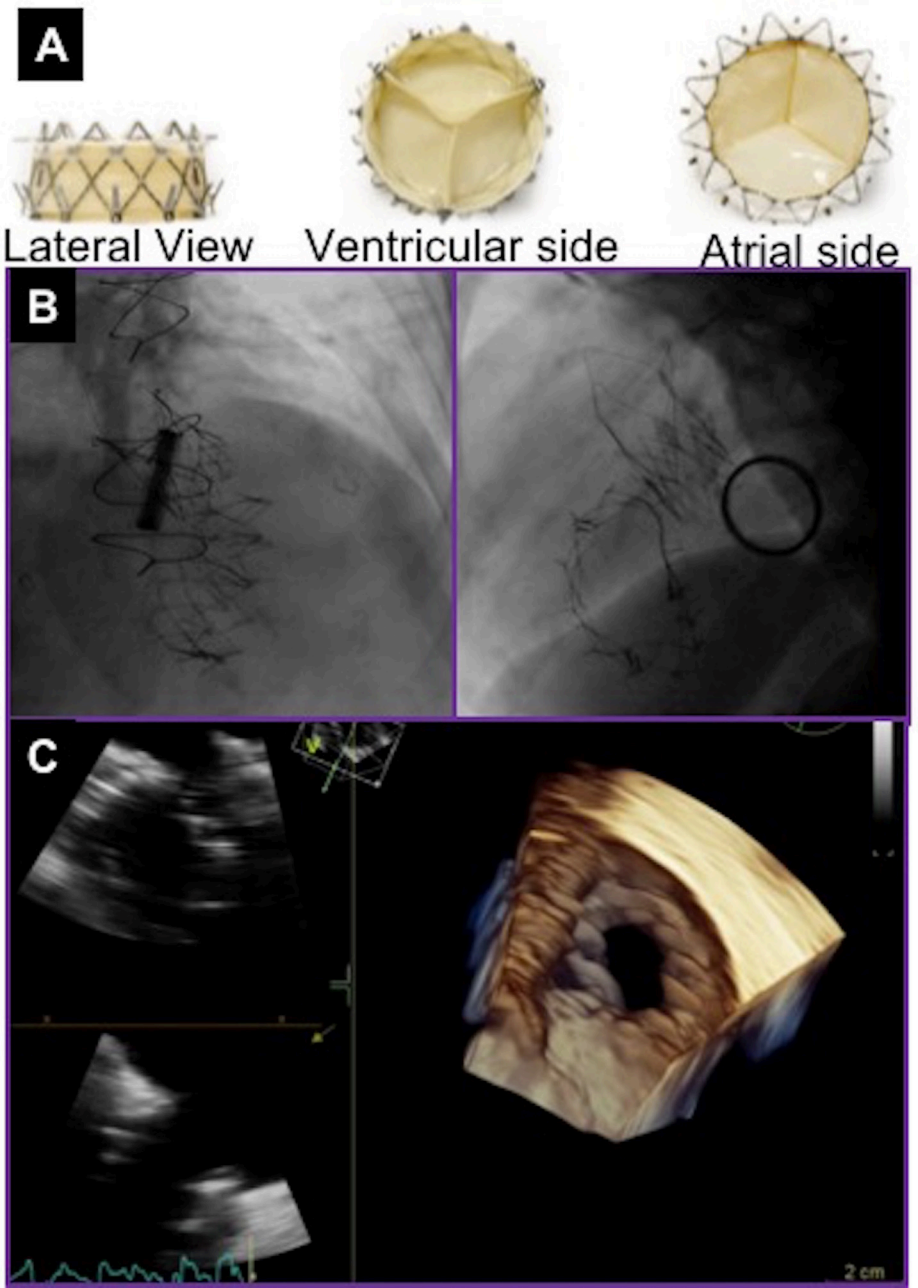
NaviGate valve and final result after valve deployment. **(A)** NaviGate Valve Profile. **(B)** Fluoroscopic images of NaviGate deployed in tricuspid annulus with relative relations with mitral valve (previous mitral valve replacement) and aortic valve (previous transcatheter aortic valve implantation). **(C)** Echocardiographic images showing good expansion and stable position of the NaviGate Valve.

NaviGate stent-valve (NaviGate Cardiac Structures, Inc, Lake Forest, CA) is a dedicated atrioventricular valve for the treatment of mitral and tricuspid regurgitation. It is a novel self-expanding valved-stent designed to treat functional tricuspid regurgitation and is available in sizes from 36 to 52 mm (Figure 4). It consists of a specifically configured Nitinol alloy stent into which is mounted a tri-leaflet valvular mechanism fabricated from equine pericardium. The configuration of the stent is specifically designed in a geometry that engages the TV annulus and TV leaflets from both inferior and superior aspects and maintains a minimal extension into both the atrium and ventricle to avoid flow dynamics alterations. Thus, the inferior aspect or ventricular diameter is designed to match the dilated TV annulus typical of secondary TR (27).

Initial pre-clinical feasibility studies demonstrated that the NaviGate is safe and feasible, and results in a secure and stable engagement of the native annulus, with excellent hemodynamic and valve performance. There have been 27 cases of first-in-human successful implantation of the NaviGate prosthesis using the transjugular and transatrial approaches. The device size implanted were 36mm in 5%, 40mm in 5%, 44mm in 27%, 48mm in 27%, and 52mm in 36% of patients. Implantation of the NaviGate TTVR resulted in TR severity reduction from severe/torrential in all patients to ≤2+ in all patients, 78% having none/trivial postprocedurally.

An important consideration is that device oversizing by 5–10% to the TV annulus or prior TV ring size to achieve better seal (9). To date, the NaviGate prosthesis has been implanted for symptomatic severe functional TR secondary to annular dilatation.

### Trisol prosthesis

**Figure 5:**
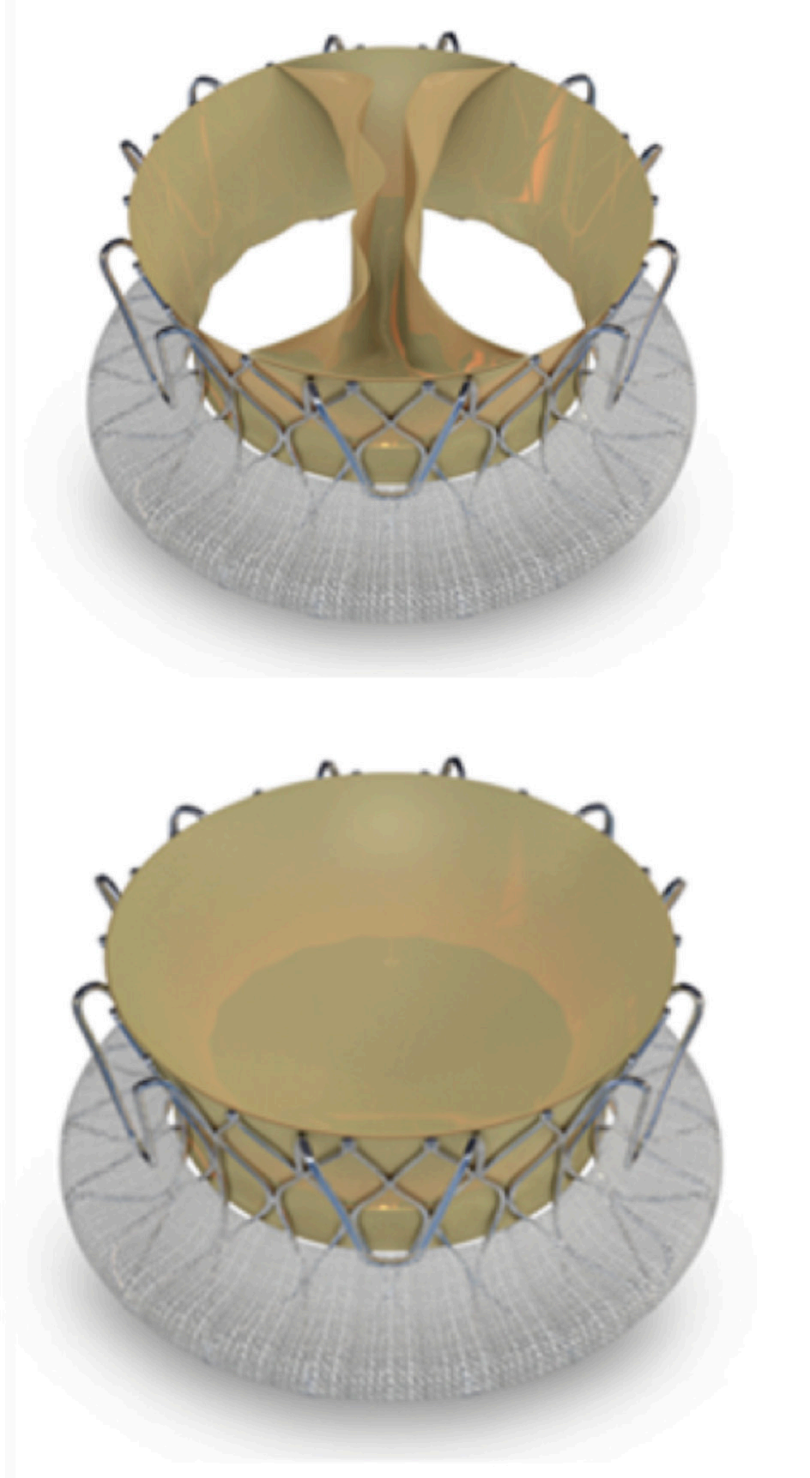
Trisol valve. Elastic nitinol frame and an inner valve apparatus.

The Trisol valve (TriSol Medical Ltd., Inc., Yokneam, Israel) is a novel percutaneous transcatheter valve representing a new concept in the treatment of severe TR (Figure 5). The TriSol valve is assembled as elastic nitinol frame and an inner valve apparatus. The nitinol frame is anchored to the annulus by multiple arms which are designed to secure the bioprosthesis into the native TV leaflets. An outside skirt seals the valve and prevents paravalvular leak. Anchoring with axial forces allows stability without affecting the conducting system. The valve apparatus is designed as a single bovine pericardial piece, attached to the nitinol frame in two opposite central commissures, and functions as two separate leaflets. The leaflets move to the center of the lumen during diastole, enabling two large lumens for the diastolic filling of the RV. During systole, the two leaflets close and coapt to the full circumference of the tricuspid annulus. The leaflets close in a dome shape structure that increases the RV closing volume to about 20ml. This increased closing volume is expected to prevent the acute surge in afterload, and to better accommodate the concomitant RV dysfunction.

### Tricuspid prostheses in development

**Figure 6:**
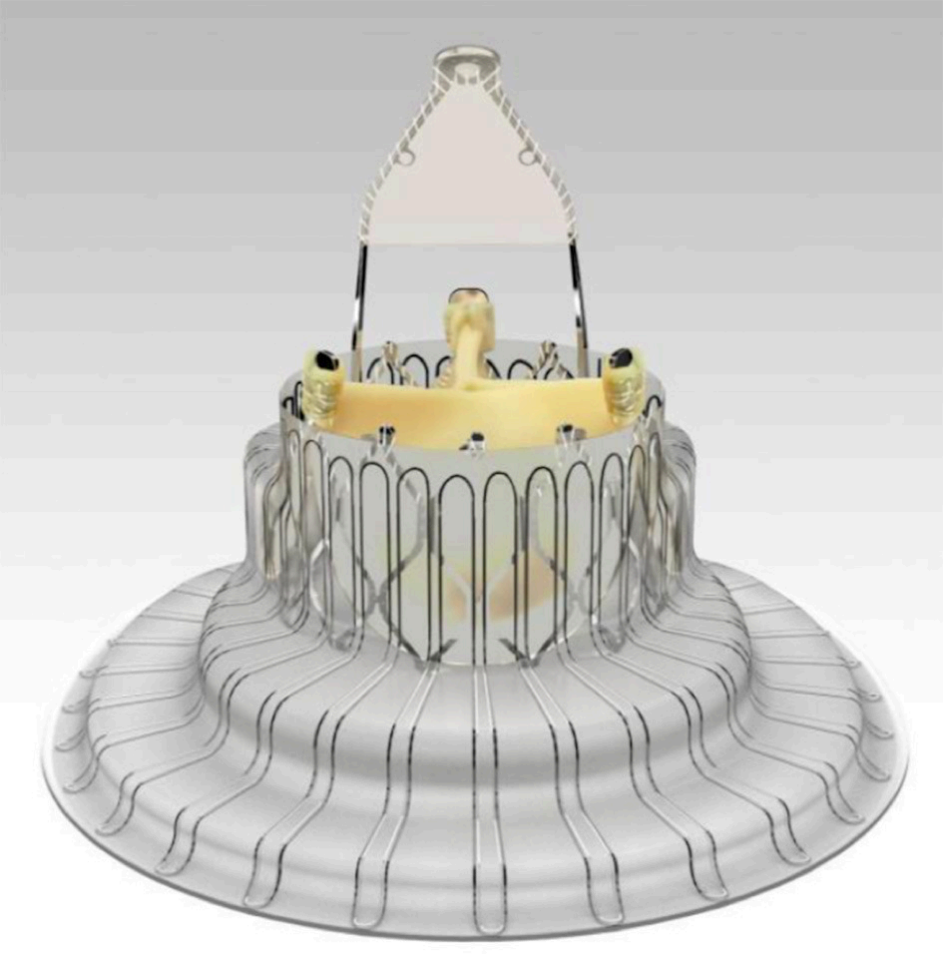
LUX-Valve. Self-expanding bovine pericardial tissue mounted on a nitinol stent frame.

**Figure 7:**
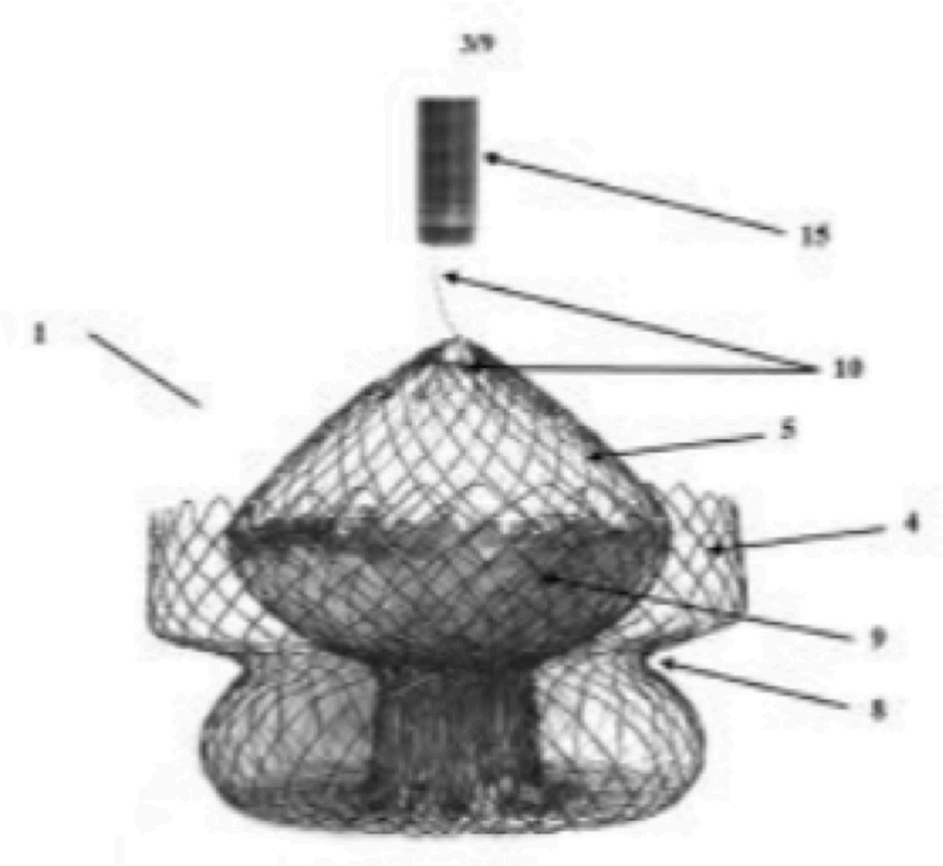
Tri-cares valve. Schematic diagram of self-expanding prosthesis made from bovine pericardial tissue mounted on a nitinol stent frame.

The LUX-valve is a Chinese designed and manufactured self-expanding prosthesis made from bovine pericardial tissue mounted on a nitinol stent frame (Figure 6). It is implanted via the right atrium and the skirt is made from self-adaptive material to minimize paravalvular regurgitation. At present successful implantation has only been demonstrated in animals. First-in-man study on the feasibility and safety of the LUX-valve is awaited. The Tri-cares (TRiCares GmbH, München, Germany) valve is a self-expanding prosthesis made from bovine pericardial tissue mounted on a nitinol stent frame (Figure 7). First-in-man study on the feasibility and safety of the Tri-cares valve is awaited.

## Conclusion

Tricuspid regurgitation is a common condition in the general population and when of moderate-to-severe is associated with poor clinical outcomes. There is an unmet clinical need for intervention in these patients. Tricuspid valve replacement is an alternative therapeutic option for these patients. We are currently at an early stage of TTVR therapies for TR and expect this field to mature significantly in the next few years. Clinical studies with new TTVR devices will enable us to elucidate which patient population will benefit the most from TTVR.

## Author contributions

OD, DR, GW, amd AL conception and design or analysis and interpretation of data, or both; OD, DR, AM, MA, SM, GW, AC, and AL: drafting of the manuscript or revising it critically for important intellectual content; OD and AL final approval of the submitted manuscript.

### Conflict of interest statement

The authors declare that the research was conducted in the absence of any commercial or financial relationships that could be construed as a potential conflict of interest. The reviewer PD declared a shared affiliation, with no collaboration, with one of the authors, AL, to the handling editor at time of review.
